# Metabolites mediate the causal associations between gut microbiota and NAFLD: a Mendelian randomization study

**DOI:** 10.1186/s12876-024-03277-w

**Published:** 2024-07-31

**Authors:** Chen Ouyang, Pengpeng Liu, Yiwei Liu, Jianwei Lan, Quanyan Liu

**Affiliations:** Department of Hepatobiliary Surgery, Tianjin Medical University General Hospital, Tianjin, 300052 P.R. China

**Keywords:** Mendelian randomization, Gut microbiota, Blood metabolite, Nonalcoholic fatty liver disease

## Abstract

**Background:**

Although gut microbiota and serum metabolite composition have been observed to be altered in patients with non-alcoholic fatty liver disease (NAFLD), previous observational studies have demonstrated inconsistent results. As this may be influenced by factors such as confounders and reverse causality, we used Mendelian randomization to clarify the causal effect of gut microbiota and blood metabolites on NAFLD.

**Methods:**

In this research, we performed a two-step Mendelian randomization analysis by utilizing genome-wide association study (GWAS) data obtained from MiBioGen and UK Biobank. To mitigate potential errors, we employed False Discovery Rate (FDR) correction and linkage unbalanced regression (LDSC) analysis. Sensitivity analyses including cML-MA and bidirectional Mendelian randomization were performed to ensure the robustness of the results.

**Results:**

In this study, a total of nine gut microbiota and seven metabolites were found to be significantly associated with NAFLD. MR analysis of the above findings revealed a causal relationship between Ruminococcus2 and cysteine-glutathione disulfide (OR = 1.17, 95%CI = 1.006–1.369, *P* = 0.041), as well as 3-indoleglyoxylic acid (OR = 1.18, 95%CI = 1.011–1.370, *P* = 0.036). For each incremental standard deviation in Ruminococcus2 abundance, there was a corresponding 26% reduction in NAFLD risk (OR = 0.74, 95%CI = 0.61–0.89, *P* = 0.0012), accompanied by a 17% increase in cysteine-glutathione disulfide levels (OR = 1.17, 95%CI = 1.01–1.37, *P* = 0.041) and an 18% increase in 3-indoleglyoxylic acid levels (OR = 1.18, 95%CI = 0.81-1.00, *P* = 0.036). The proportion mediated by cysteine-glutathione disulfide is 11.2%, while the proportion mediated by 3-indoleglyoxylic acid is 7.5%.

**Conclusion:**

Our study suggests that increased abundance of specific gut microbiota may reduce the risk of developing NAFLD, and this relationship could potentially be mediated through blood metabolites.

**Supplementary Information:**

The online version contains supplementary material available at 10.1186/s12876-024-03277-w.

## Introduction

Non-alcoholic fatty liver disease (NAFLD) is a prevalent liver ailment that affects a large section of the world’s population, estimated to be around 32.4% [1]. It is defined by an overabundance of triglycerides accumulating in hepatocytes, which can cause organ damage and hepatic inflammation. Due to its rising prevalence and close relationship to a variety of metabolic conditions, including obesity, type 2 diabetes mellitus, and dyslipidemia, NAFLD has emerged as a significant public health concern [2]. Furthermore, NAFLD imposes a substantial economic burden on healthcare systems worldwide owing to its associated complications including cirrhosis, hepatocellular carcinoma (HCC), and cardiovascular diseases. This prevalent metabolic disorder exhibits multifactorial etiology encompassing insulin resistance, metabolic syndrome, genetic predisposition, lifestyle choices, and levels of physical activity [3].

Extensive research conducted in the last few years has shed light on the complex association between gut microbiota dysbiosis and the development of NAFLD [4]. The gut microbiome is a complex ecosystem comprising diverse microorganisms, including bacteria, viruses, fungi, archaea, bacteriophages, and protozoa that reside within the gastrointestinal tract [5]. Dysregulation of the gut microbiota can lead to the disturbance of intestinal mucosal barrier function through liver-gut axis [6]. The gut mucosa acts as a protective barrier against harmful compounds in diets, keeping them from entering the bloodstream. However, gut microbiota dysbiosis can compromise this barrier, leading to the translocation of bacterial metabolites and other toxic substances from the intestine into systemic circulation. This process is commonly referred to as ‘leaky gut’ [7]. The impairment of the intestinal barrier function not only allows harmful substances to directly expose hepatocytes but also initiates an inflammatory response in both the intestines and livers. This inflammation accelerates liver damage by elevating insulin resistance and oxidative stress, both of which are significant factors in the development of NAFLD.

Metabolites are tiny chemicals that are essential components of the body’s intricate metabolic system. These compounds serve as intermediates or end products in various biochemical pathways that are indispensable for maintaining normal physiological functions. Emanuel et al. demonstrated that the gut microbiota can breakdown indigestible carbohydrates and produces metabolites related to NAFLD including short-chain fatty acids and succinate [8, 9]. Despite evidence highlighting the impact of gut microbes on NAFLD through metabolite production, our current understanding is limited to a subset of these metabolites. Given the large number of metabolites produced by the gut flora, further comprehensive studies are necessary on those metabolites specifically associated with NAFLD. Moreover, previous studies still face potential challenges related to confounding variables and reverse causality.

Mendelian randomization (MR) is a widely used approach in biomedical research that employs genetic variation to investigate potential causal connections between exposures and outcomes. It offers a promising option for addressing the issues of confusion and reverse causality [10]. Genome-wide association studies (GWAS) have recently made considerable advancements in identifying the genetic factors that influence the human metabolome [11]. The study presented GWAS data for 1091 blood metabolites and 309 other metabolites. The main purpose of this study was to perform a comprehensive MR analysis of several large datasets to identify metabolites that may play a mediating role between gut microbiota and NAFLD.

## Materials and methods

### Study design

All datasets utilized in this study are available on the database website. The institutional review boards’ ethics committees had approved written informed consent for all participants, eliminating the requirement for further ethical approval or informed consent.

This study adopted a two-sample, two-step MR strategy that included summary data from GWAS. This study satisfies the STROBE-MR criteria and strictly follows three basic assumptions when selecting instrumental variables (IVs) [12], include: (1) Strong correlation between IVs and the exposure under investigation; (2) Independence of IVs from any known or unknown confounding factors; (3) Sole influence of IVs on outcomes through risk factors without involvement in any other direct causal pathway. To ensure sample independence, we selected metabolites, gut microbiota, and NAFLD from distinct GWAS datasets for this study.

**Fig. 1:**
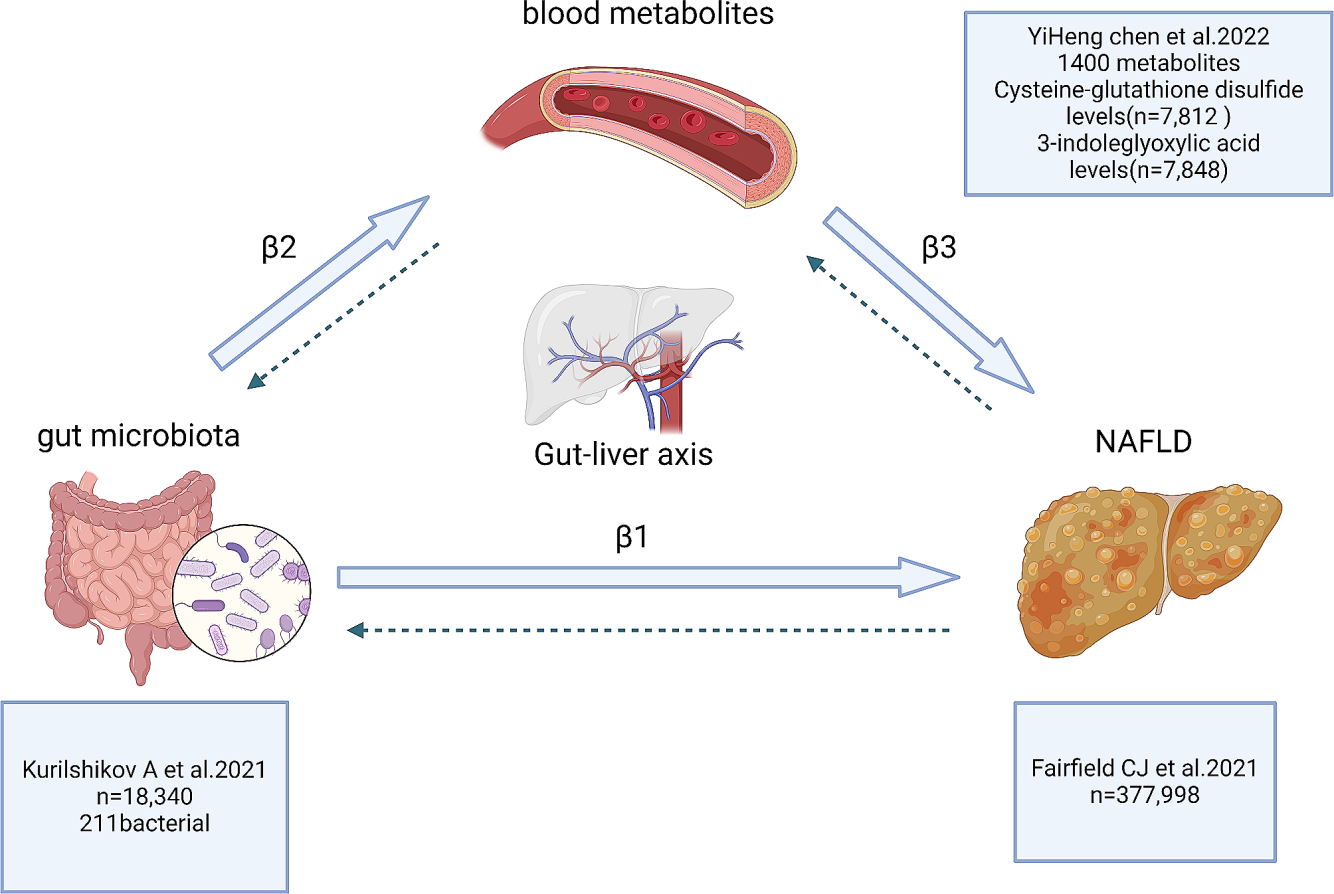
Schematic study design showing the specific experimental design of this study. Demonstration of the connection between NAFLD, blood metabolites, and gut microbiota via the liver-gut axis. Dotted line indicates inverse MR analysis

In this investigation, we performed a two-step MR analysis. Initially, the causal relationship between gut microbiota and NAFLD was assessed by two-sample MR analysis and an effect size β1 was calculated. Subsequently, an additional MR analyze was conducted to examine the causal connection between NAFLD and a comprehensive set of 1400 metabolites. The obtained effect size β3 underwent multiple p-value corrections. Ultimately, we carried out MR analyses on the gut microbiota and metabolites to determine the impact size of β2. Figure 1 depicts the schematic structure of this research design.

### Data sources

The gut microbiota data for this study were obtained from the MiBioGen consortium’s comprehensive GWAS meta-analysis (https://mibiogen.gcc.rug.nl/). This most comprehensive study to date utilized 16 S rRNA technology to analyze the genetic diversity of 211 gut microbiome species in 18,340 individuals from 24 different groups [13]. Among the participants, a total of 13,266 individuals were identified as having European heritage.

The data summary for NAFLD was obtained from the IEU GWAS project (https://gwas.mrcieu.ac.uk/), and the GWAS ID was ebi-a-GCST90054782. In this investigation, scientists performed a case-control GWAS using the UK Biobank dataset and relied on documented diagnoses of NAFLD according to diagnostic codes suggested by recent consensus guidelines. The NAFLD summary statistics encompassed 377,988 individuals of European ancestry, comprising 4,761 NAFLD patients and 373,227 controls. For further details regarding these GWAS data, referring to the primary literature sources is advised [14].

The GWAS data for the 1400 metabolites were obtained from Chen et al. [11], which is the most comprehensive analysis of blood metabolites using GWAS data so far. This study involved 8,299 people of European ancestry who were genotyped and had their circulating plasma metabolites measured. Table 1 lists the sources of all data for this study, and specific download links for the 1400 metabolites are provided in Supplementary Table 1.

**Table 1:** An overview of the data and download links for this research

Reported trait	GWAS ID	Sources of data	Sample size	Online resource
Gut microbiota	PMID: 33,462,485	MiBioGen consortium	18,340 European ancestry	https://mibiogen.gcc.rug.nl/
NAFLD	ebi-a-GCST90054782	IEU open gwas	377,998 European ancestry	https://gwas.mrcieu.ac.uk/
1400 metabolites	GCST90199621-GCST90201020	GWAS Catalog	8,299 European ancestry	https://www.ebi.ac.uk/

### Selection of instrumental variables (IVs)

To ensure the accuracy of our results, we applied a meticulous screening process to the gut microbiota we collected. Firstly, based on previous MR studies, we employed a significance threshold of *p* < 1E-05 for the screening of single nucleotide polymorphisms (SNPs) [15]. Screening for statistically significant SNPs associated with 211 gut microbiota species was conducted. Additionally, to mitigate the influence of genetic pleiotropy on the findings, a chain disequilibrium coefficient r2 = 0.001 and a region width of 10,000 kb were employed [16]. Furthermore, the presence of weak instrumental bias was assessed by calculating the F-statistic (F = Beta^2^/SE^2^), whereby an F value greater than 10 indicates its absence [17].

### Mendelian randomization analysis

In order to conduct a thorough investigation and determine the cause-and-effect relationship between the gut microbiota and NAFLD, five reliable methods were employed in this study: MR Egger, weighted median (WM), inverse variance weighted (IVW), simple mode, and weighted mode. The weighted median method is a central trend estimator, and causality can be calculated in the presence of confounders as long as more than half of the valid IVs are available [18]. Since the IVW approach can establish causation by a comprehensive meta-analysis of Wald scores, it was chosen as the main strategy [19]. Additionally, we utilized MR-egger regression to address potential horizontal pleiotropy. The MR-Egger technique operates on the assumption that the validity factors are independent, suggesting no connection between the effect of genetic variation on the outcome variable and its effect on the exposure variable [20]. The IVW methodology was mainly used in this work, with other approaches implemented as supplemental analyses to enhance and support the IVW method.

### Sensitivity analysis

We performed various sensitivity analyses to assess the statistical significance of the findings. Initially, we evaluated the genetic connections between metabolites and NAFLD via Linkage Disequilibrium Score Regression (LDSC) analysis. Previous research has indicated that the presence of genetic correlations between traits can lead to false positive Mendelian randomization results, even after removing SNPs associated with NAFLD. LDSC enables the assessment of genetic correlation between polygenic characteristics by leveraging the linkage disequilibrium patterns among common genetic variations in GWAS summary data [21]. Hence, genetic correlations between metabolites and NAFLD were evaluated using LDSC to ascertain if causal effects are influenced by shared genetic architecture. In the LDSC analysis, a threshold of *p* < 0.007 (adjusted by Bonferroni correction) was utilized to establish statistical significance, aiming to minimize false positive results. Additionally, False discovery rate (FDR) correction of p-values was employed in the MR analysis of multiple metabolites and NAFLD. The FDR correction is a widely employed statistical method utilized to mitigate the effects of multiple comparisons and efficiently control error rates. In this study, correlations were considered significant when *p* < 0.05 and q < 0.2 [22]. Subsequently, we conducted a sensitivity analysis that included Cochrane’s Q test and MR-Egger regression. The heterogeneity of instrumental variables was assessed by Cochrane’s Q test, which indicates heterogeneity among the IVs when *P* < 0.05. We used MR-Egger regression to investigate the potential existence of pleiotropy, which could be ruled out if the intercept’s p-value exceeded 0.05 [23]. Furthermore, we utilized the most recent cML-MA technique to mitigate the potential bias stemming from correlation level pleiotropy [24].

### Reverse mendelian randomization

All causal relationships in this study were validated by reverse Mendelian randomization analysis, whose main purpose is to reduce the effect of reverse causality.

The R software (version 4.3.2) was used to conduct the statistical analysis and data visualizations described above. It involved freely accessible packages including “TwoSample MR”, “MRPRESSO”, “MRcML” and “ldscr”.

## Results

### Selection of instrument variables

Initially, we conducted a rigorous filtering process on GWAS data for 211 gut microbiotas (*n* = 18,304), applying stringent criteria of SNP screening at the level of *P* < 1E-05 and ensuring that the F-statistic for all SNPs exceeded 10. This meticulous method allowed us to minimize any potential bias caused by weak instrumental factors, resulting in a strong association between SNPs and gut microbiota in our research. Subsequently, we successfully identified instrumental variables related to 1400 metabolites with a significance level of *P* < 1E-05. Instrumental variables after screening for metabolites are shown in Supplementary Table 2. The Supplementary Tables 3 and Supplementary Table 4 list the instrumental variables screened for gut microbiota.

### Causal relationships between gut microbiota and NAFLD

**Fig. 2:**
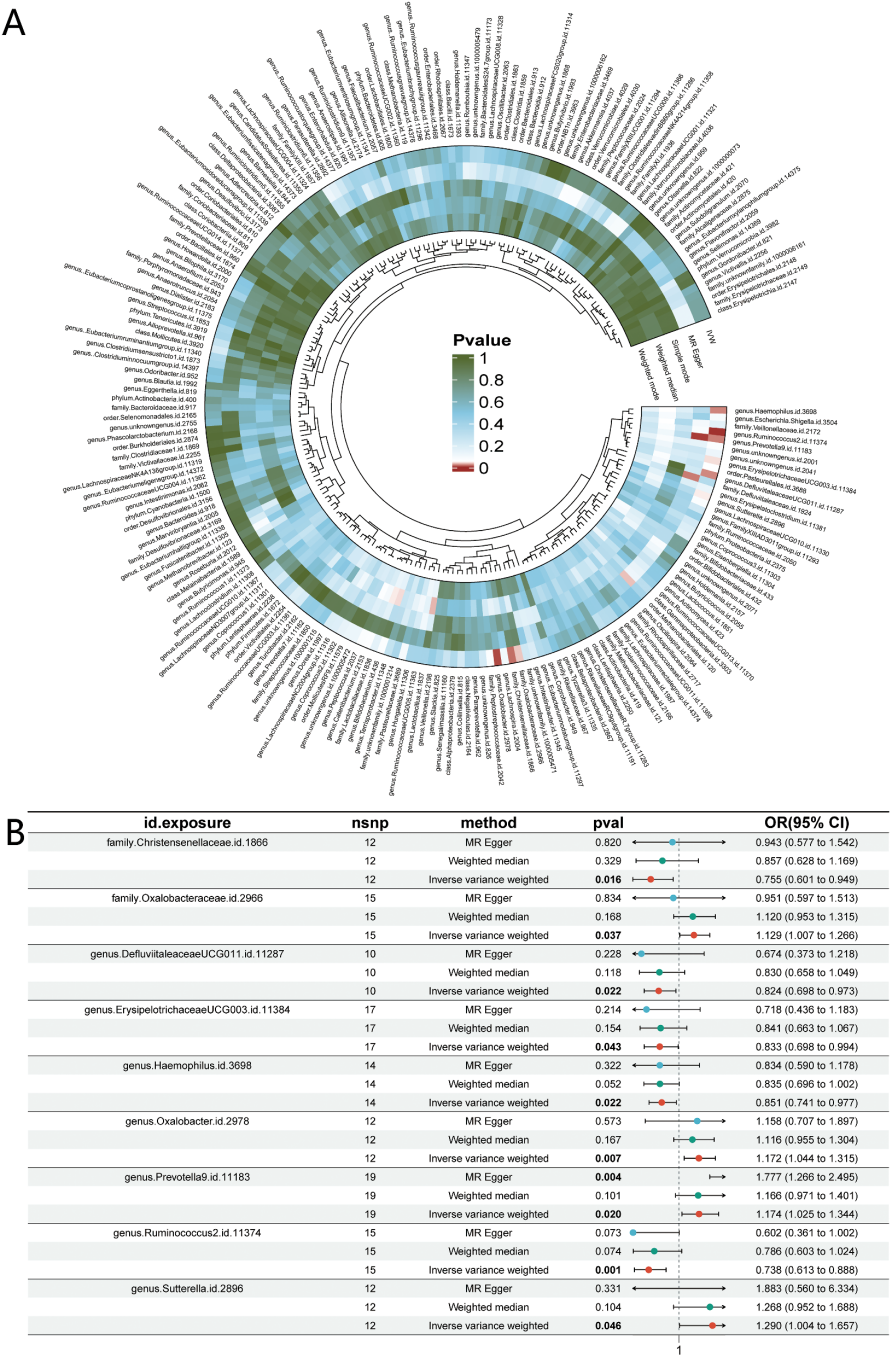
MR evaluation of NAFLD patients’ gut microbiome. (**A**) The P values for IVW, MR Egger, simple mode, weighted median, and weighted mode are shown, in that order, from outside to inside. (**B**) A forest plot showing the MR data between NAFLD and nine gut microbiotas

After filtering SNPs affected by linkage palindrome structure and linkage disequilibrium, a total of 10 to 19 SNPs remained for further analysis. Utilizing the IVW method, we identified nine gut microbiota abundances that are genetically causally associated with NAFLD. The results of the MR analysis are presented in Fig. 2. Notably, Ruminococcus2 (OR = 0.74, OR95%CI = 0.61–0.89, P-value = 0.001) demonstrated a significant correlation indicating that genetically predicted Ruminococcus2 might potentially reduce the risk of developing NAFLD. Sensitivity analyses employing Cochran’s Q test and MR-Egger methods did not reveal any indications of pleiotropy (*P* > 0.05). No horizontal pleiotropy was found during analysis, the cML-MA method indicates the absence of vertical pleiotropy (*P* < 0.01). By two-sample MR analysis, we calculated an effect size (β1) of -0.30 between Ruminococcus2 and NAFLD. Table 2 displays the findings of the sensitivity analysis, which did not provide any indication of heterogeneity among the NAFLD-associated gut microbiota. The MR-Egger test showed that Prevotella9 and Oxalobacter had horizontal pleiotropy (Table 2), so we used the MR-Egger result as the main MR analysis method instead of the IVW method. And a significant causal relationship between Prevotella9 (*P *= 0.004) and NAFLD was suggested by the MR-egger result. No abnormal SNPs were found in the leave-one-out analysis, indicating that individual SNPs do not influence MR results (Supplementary Fig. 1). Figure 4 A illustrates the analytical framework for this analysis.

**Table 2:** Results of sensitivity analysis between gut microbiotas and NAFLD

Outcome	Exposure	Heterogeneity		MR-egger		cML-MA
		Cochran’s Q	*P*	Intercept value	*P*	*P*
NAFLD	Christensenellaceae	12.5	0.25	-0.018	0.34	0.0072
NAFLD	Oxalobacteraceae	14.4	0.35	0.024	0.47	0.028
NAFLD	DefluviitaleaceaeUCG011	6.65	0.58	0.022	0.51	0.022
NAFLD	ErysipelotrichaceaeUCG003	17.54	0.29	0.014	0.54	0.029
NAFLD	Haemophilus	4.15	0.99	0.0023	0.9	0.022
NAFLD	Oxalobacter	9.29	0.6	0.0018	0.036	0.0068
NAFLD	Prevotella9	20.04	0.33	-0.044	0.019	0.016
NAFLD	Ruminococcus2	13.47	0.49	0.016	0.42	0.0013
NAFLD	Sutterella	16.75	0.12	-0.026	0.55	0.013

Afterwards, we proceeded with a reverse MR analysis, utilizing the seven gut microbiotas associated with NAFLD as outcomes and NAFLD itself as the exposure. However, no substantial evidence was found to support the causal effects of NAFLD on the aforementioned microbial taxa. The instrumental variables and results of inverse Mendelian randomization can be found in Supplementary Tables 5 and Supplementary Table 6, respectively.

### Causal relationships between 1400 metabolites and NAFLD

**Fig. 3:**
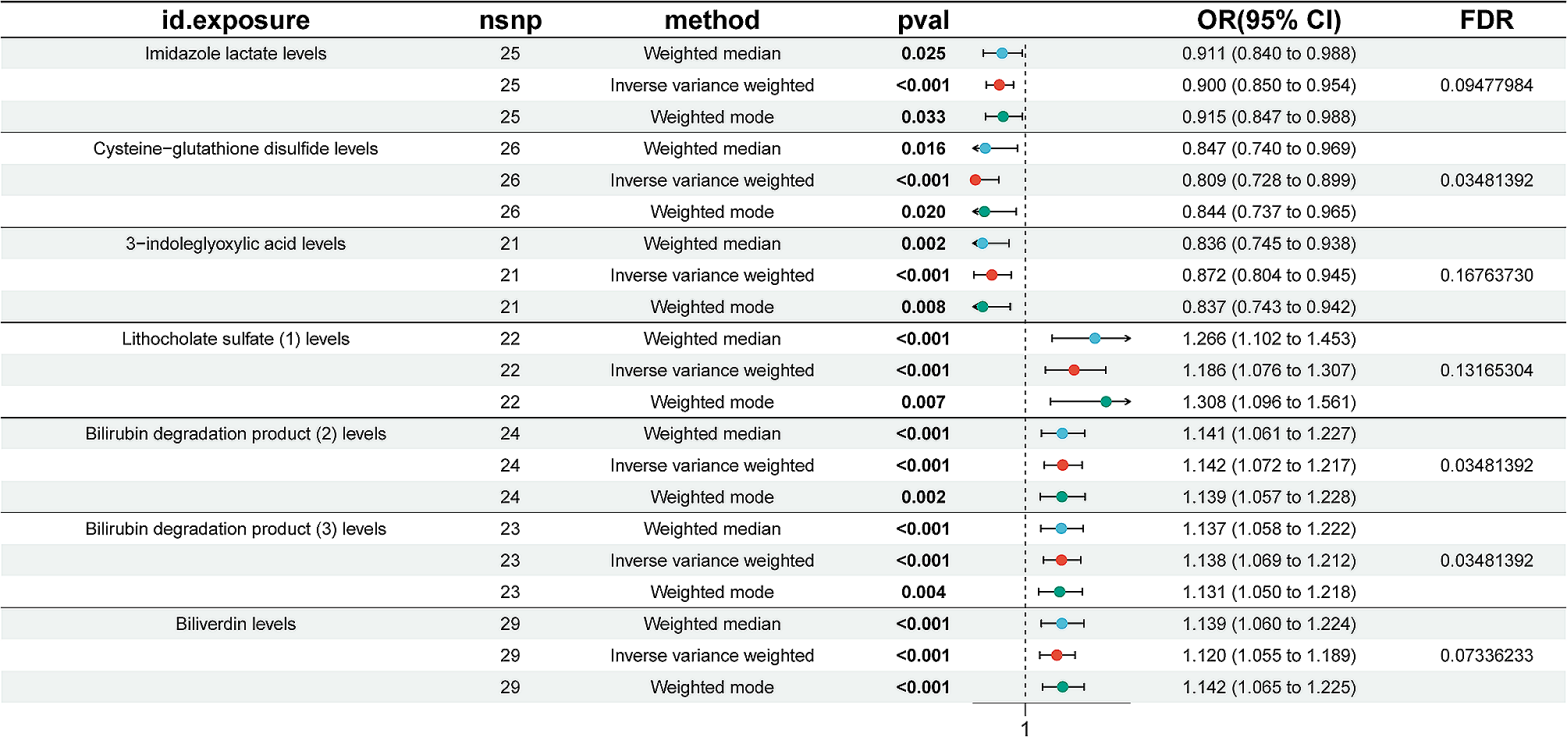
Forest plot depicting the effect of metabolite causality on the probability of NAFLD after applying FDR correction based on MR data

We conducted separate univariate MR analysis on 1400 metabolite levels in NAFLD patients. Multiple hypothesis testing was performed throughout the association analysis of metabolites and their association with NAFLD. To minimize the likelihood of false positives, we employed the FDR method with a threshold set at q < 0.2 Seven metabolites exhibited a significant causal relationship with NAFLD following FDR correction, specifically Imidazole lactate (OR = 0.90, *P* = 0.00035, FDR = 0.094), Cysteine-glutathione disulfide(OR = 0.81, *P* = 0.000078, FDR = 0.035), 3-indoleglyoxylic acid(OR = 0.87, *P* = 0.00088, FDR = 0.16), Lithocholate sulfate [1] (OR = 1.19, *P* = 0.00059, FDR = 0.13), Bilirubin degradation product-C17H18N2O4 [2] (OR = 1.14, *P* = 0.00004, FDR = 0.035), Bilirubin degradation-C17H18N2O4 [3] (OR = 1.14, *P* = 0.00005, FDR = 0.035)and Biliverdin (OR = 1.12, *P* = 0.0002, FDR = 0.07). The results of univariable MR analyses for each metabolite associated with NAFLD are presented in Fig. 3. The estimation orientations of the three approaches (MR-Egger, IVW and WM) exhibited concurrence. Subsequently, sensitivity assessments were then carried out. Cochran’s Q test, MR-Egger analysis, and MRPRESSO results all indicate a lack of pleiotropy (*P* > 0.05), as shown in Supplementary Table 7. Leave-one-out analysis suggests that causality is not influenced by a single SNP (Supplementary Fig. 2). The genetic correlation between NAFLD and these seven metabolites was then assessed using an LDSC analysis, the results are presented in Supplementary Table 8. According to the LDSC approach, there was no discernible genetic association between NAFLD and levels of 3-indoleglyoxylic acid (rg = 0.011, *P* = 0.95), Lithocholate sulfate [1] (rg = 0.61, *P* = 0.015), Bilirubin degradation product C17H18N2O4 [2] (rg=-0.041, *P* = 0.91), Bilirubin degradation product C17H18N2O4 [3] (rg=-0.041, *P* = 0.87), or Biliverdin (rg = 0.26, *P* = 0.29). However, there was a statistically significant association between imidazole lactate (rg = 0.58, *P* = 0.0058) and cysteine-glutathione disulfide (rg=-0.55, *P* = 1.53E-05) with NAFLD. This suggests that the observed MR causality mentioned above may be confounded by a shared genetic structure, potentially arising from a mismatch between the GWAS samples and the reference dataset employed for LDSC analysis [25]. This mismatch introduces inaccuracies that lead to less precise estimations.

**Fig. 4:**
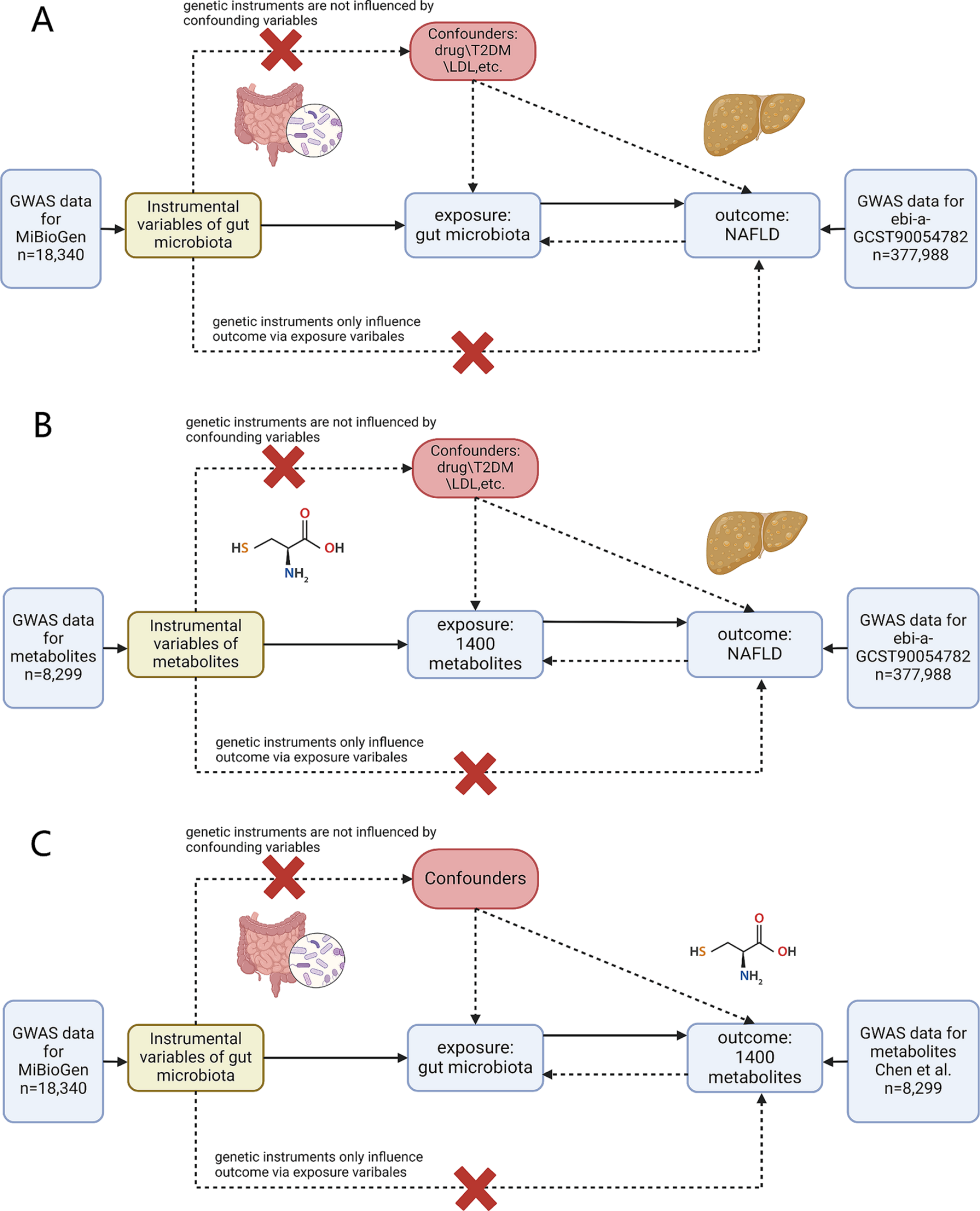
Detailed flowchart designed for Mendelian randomization analysis, we employed bidirectional MR method in each step (**A**) Evaluate the potential relationship between gut microbiota and NAFLD. (**B**) Investigate the causal relationship between different metabolites and NAFLD. (**C**) Examine the causal link between gut microbiota and metabolites

We subsequently performed a reverse MR analysis utilizing seven metabolites that exhibited genetic associations with the risk of NAFLD as outcome. Interestingly, our reverse MR analysis revealed significant effects of NAFLD on three specific metabolites: biliverdin and two Bilirubin degradation products (C17H18N2O4 [2], C17H18N2O4 [3]). NAFLD could increase the serum level of the Bilirubin degradation product-C17H18N2O4 [2] (OR = 1.07, *P* = 0.047), Bilirubin degradation product-C17H18N2O4 [3] (OR = 1.08, *P* = 0.013), and Biliverdin (OR = 1.07, *P* = 0.038). Considering the presence of reverse causality, these three metabolites were excluded from subsequent analyses. The IVs utilized in the reverse MR analysis were presented in Supplementary Table 5, while the corresponding outcomes were displayed in Supplementary Table 9. MR analysis results of other metabolites with NAFLD were not affected by reverse causality. The analytical framework is depicted in Fig. 4B.

Because no bias was introduced during the analysis and there were no duplicate instrumental variables in exposure and mediation, we did not perform multivariate MR analyses and utilized the four metabolites mentioned above as mediators for subsequent analyses [16]. The univariate MR analysis yielded effect sizes (β3_cdg_=-0.21, *P* < 0.01) indicating a causal relationship between Cysteine-glutathione disulfide level and NAFLD. Similarly, the effect size between 3-indoleglyoxylic acid level and NAFLD was found to be β3_i.a._=-0.14, *P* < 0.01.

### Causal relationships between gut microbiota and metabolites

After identifying the gut microbiota and metabolites associated with NAFLD, we conducted MR analysis on these factors. Utilizing the IVW method, we discovered that levels of Cysteine-glutathione disulfide (OR = 1.17, 95%CI = 1.006–1.369, *P* = 0.041) and 3-indoleglyoxylic acid (OR = 1.18, 95%CI = 1.011–1.370, *P* = 0.036) were significantly causally linked to Ruminococcus2. Furthermore, the level of lithocholate sulfate [1] was causally associated with Prevotella9 (OR = 0.90, 95%CI = 0.810–0.995, *P* = 0.041). The results of the IVW analysis are presented in Supplementary Table 10. Cochran’s Q test and MR-Egger calculated p-values greater than 0.05, indicating the absence of horizontal pleiotropy and heterogeneity. In addition, the cML-MA test (*P* < 0.05) demonstrated the absence of horizontal or vertical pleiotropy (Supplementary Table 11). Leave-one-out analysis suggested that no single SNP modified the observed causal associations (Supplementary Fig. 3). The univariate MR analysis revealed a significant effect size of β2_cdg_ = 0.16 for the causal relationship between Ruminococcus2 and Cysteine-glutathione disulfide level. And the effect size between Ruminococcus2 and 3-indoleglyoxylic acid was β2_i.a._ = 0.16. Similarly, we calculated an effect size of β2_ls_=-0.11 for the association between Prevotella9 and Lithocholate sulfate [1] level. The analytical framework for this step was presented in Fig. 4c.

Afterwards, we conducted a reverse MR analysis using the three metabolites and their associated gut microbiotas. Given the limited number of SNPs detected at a significance threshold of *P* = 5E-08, we decided to adopt a more lenient threshold of *P* = 5E-06 for this reverse MR analysis. The results of reverse MR indicated the absence of reverse causality in our analysis. Supplementary Table 12 provides the SNPs utilized in the reverse MR analysis, while Supplementary Table 13 presents the corresponding results obtained from this analysis.

### Mediation effect of cysteine-glutathione disulfide and 3-indoleglyoxylic acid

Using a two-step MR analysis, we assessed the potential mediating role of Cysteine-glutathione disulfide levels in the causal association between Ruminococcus2 and the risk of NAFLD. The mediated proportion was found to be 11.2% (95%CI = 5.6–16.8%), which elucidated that for every standard deviation increase in Ruminococcus2 abundance, there was a corresponding 26% reduction in NAFLD risk, accompanied by a 17% increase in cysteine-glutathione disulfide levels. Conversely, augmenting Cysteine-glutathione disulfide levels by one standard deviation would lead to a decrease of 19% in NAFLD risk.

**Fig. 5:**
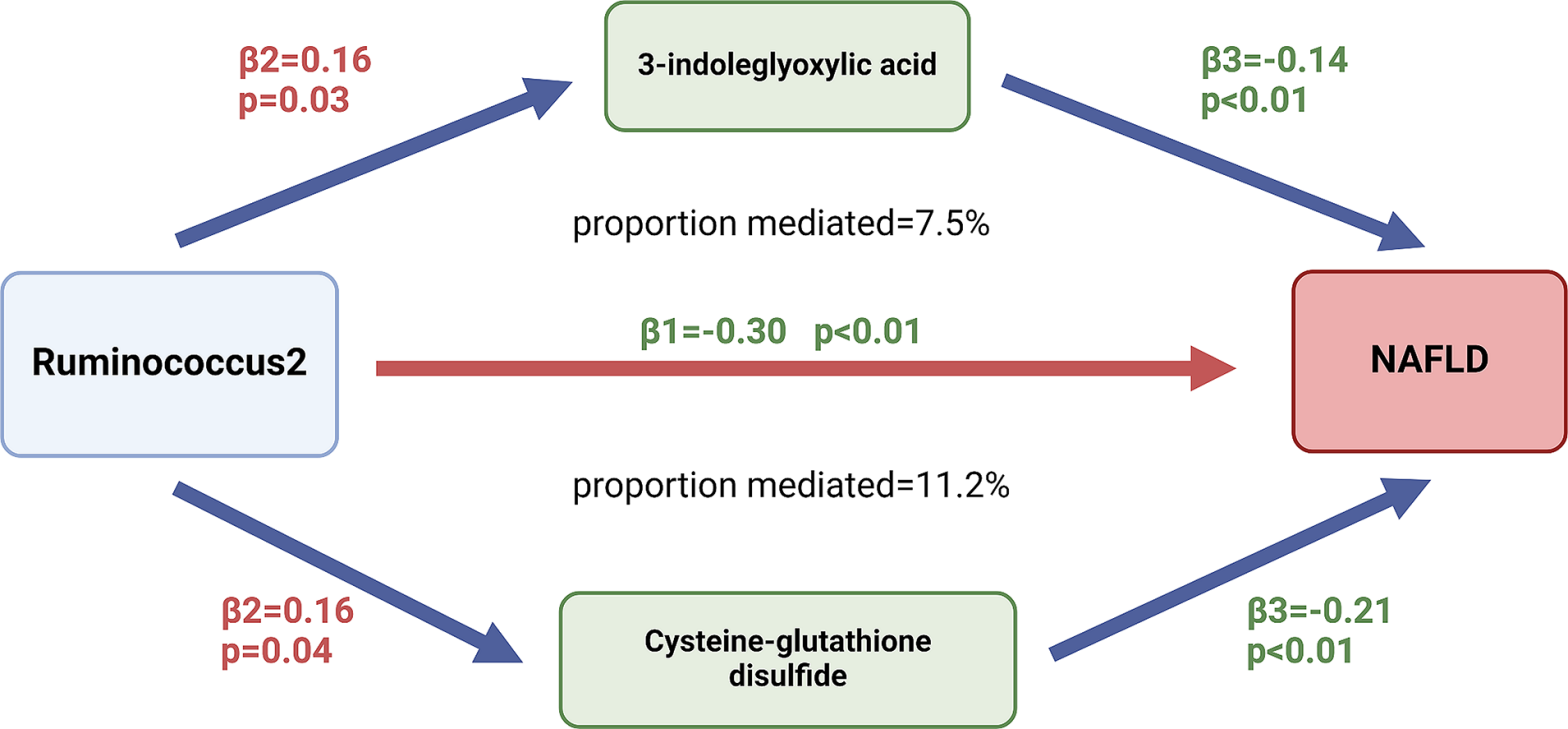
The mediating role of 3-indoleglyoxylic acid and cysteine-glutathione disulfide levels in the causal relationship between Ruminococcus2 and NAFLD is examined

Similarly, we conducted an analysis to determine the potential mediating role of 3-indoleglyoxylic acid levels (proportion mediated = 7.5%, 95%CI = 2.9–12.0%) in the relationship between Ruminococcus2 and the risk of NAFLD. Our findings suggest that for each standard deviation increase in Ruminococcus2 abundance, there is a corresponding decrease of 26% in NAFLD risk, accompanied by an 18% increase in 3-indoleglyoxylic acid levels. Furthermore, each standard deviation increase in 3-indole glycine levels was associated with a significant reduction of 13% in the likelihood of developing NAFLD. The mediation effect was illustrated in Fig. 5. However, the mediation effect did not hold when a two-step MR analysis is conducted using Lithocholate sulfate [1] levels as a mediator between Prevotella9 and NAFLD. The effect size (β2) for the causal relationship between Prevotella9 and Lithocholate sulfate [1] levels was observed to be -0.11. Additionally, the effect size between Prevotella9 and NAFLD was found to be 0.16(β1), while the effect size between Lithocholate sulfate [1] levels and NAFLD was determined to be 0.17(β3). In terms of the mediate pathway, it was discovered that increased levels of Prevotella9 lead to a decrease in serum lithocholate sulfate [1] levels, consequently reducing the risk of NAFLD. However, in contrast to these intermediate results, univariable MR analysis findings suggested that elevated levels of intestinal Prevotella9 actually increased the risk of NAFLD, indicating inconsistency with our initial hypothesis. We hypothesize that this discrepancy could potentially be attributed to the presence of a more powerful mediation in Prevotella9 compared to lithocholate sulfate, or it might be influenced by the existence of horizontal pleiotropy within Prevotella9 SNPs.

(proportion mediated=$\frac{\beta 2*\beta 3}{\beta 1}$; $S=\sqrt{{\beta 2}^{2}*{se2}^{2}+{\beta 3}^{2}*{se3}^{2}}$; 95%CI= $\frac{\beta 2*\beta 3-S}{\beta 1}$%-$\frac{\beta 2*\beta 3+S}{\beta 1}$%). In addition, scatter plots of the MR analyses are shown in Figure S4.

## Discussion

To the best of our understanding, this research represents the first comprehensive and thorough investigation of the association among gut microbiota, NAFLD, and metabolites using openly available genetic data. In this study, we combined GWAS data from multiple databases, including MiBioGen and UK Biobank, for a comprehensive two-sample MR analysis. Our findings suggest a negative association between the abundance of Ruminococcus2 in the gastrointestinal tract and the incidence of NAFLD. Furthermore, it appears that this protective effect may be mediated by an increase in cysteine-glutathione levels (proportion mediated = 11.2%) as well as 3-indoleglyoxylic acid levels (proportion mediated = 7.5%).

We notice a strong causal link between the dysregulation of Ruminococcus2 and the likelihood of developing NAFLD. Extensive evidence from numerous previous observational studies suggests that an imbalance in the gut microbiota is related with the pathogenesis of NAFLD [26], which aligns with our findings. Given that the portal venous system of the liver collects blood from the intestines, there exists a complex interdependent relationship between these two organs known as the gut-liver axis [27]. Increasing evidence supports a role for gut microbiota in driving NAFLD progression through this axis. For instance, Daniela Stols-Gonçalves et al. utilized a multi-omics approach to demonstrate that fecal microbiota transplantation (FMT) induces substantial alterations in plasma metabolites and hepatic DNA methylation profiles among patients with NAFLD [28]. In addition, recent research has shown that the gut microbiota can ferment indigestible carbohydrates, like dietary fiber, producing succinate and other essential metabolites [8]. These metabolites subsequently enter circulation and exert an impact on the progression of NAFLD. Ruminococcus2, a Gram-positive, spherical-looking anaerobic bacterium of the genus Ruminococcus, has been shown to associated with body weight indices (including waist circumference and body mass index), and lipid indices (including LDL, triglycerides and total cholesterol) [29]. Given that obesity and dyslipidemia are notable risk factors for NAFLD [30], the aforementioned studies substantiate our findings. Therefore, we hypothesize that Ruminococcus2 may produce a specific metabolite that influences the advancement of NAFLD through the gut-liver axis.

The correlation between metabolites and gut microbiota has emerged as a prominent area in contemporary research. The gut microbiota can produce metabolites via fiber fermentation, which are absorbed into the bloodstream and modulate the immune system [31]. Our study reveals that an increased abundance of Ruminococcus2 leads to elevated levels of cysteine-glutathione disulfide and 3-indoleglyoxylic acid. Currently, there are fewer experimental studies on the relationship between the gut microbiota and the levels of cysteine-glutathione disulfide. Adil Mardinoglu and colleagues’ research suggests that gut microbiota in mice regulates the metabolism of amino acids and glutathione in the host [32]. Likewise, Peipei Chen et al. have proposed that the gut microbiota undergoes alterations in mice with adenomyosis, consequently impacting the intestinal metabolite cysteine [33]. The above studies demonstrate that gut microbes affect these two metabolites separately. Cysteine-glutathione disulfide is not an endogenous metabolite; rather, it arises from the combination of cysteine residues on proteins and glutathione during oxidative stress conditions [34], and thus this evidence supports our conclusion. 3-indoleglyoxylic acid is a kind of organic chemical that originates from indole-3-acetic acid. Similar to cysteine-glutathione disulfide, there are limited investigations regarding the association between 3-indol-glyoxylic acid and gut microbiota. In essence, this suggests that these metabolites represent novel and unconventional compounds that potentially play significant roles in organisms. Recent research has shown that the gut microbiota in mice may synthesize indoleacetic acid and impact neuroinflammation. This finding partially supports our hypothesis [35].

Metabolites produced by the intestinal microbiota can potentially contribute to the pathogenesis of NAFLD through various mechanisms. Firstly, these metabolites can directly impact hepatic lipid metabolism, such as short-chain fatty acids [36]. Homocysteine and glutathione have been demonstrated to be involved in lipid metabolism [37], thus suggesting that cysteine-glutathione disulfide could potentially influence the progression of NAFLD in this manner. Secondly, disruption of the gut microbiota leads to inflammation, thereby compromising the integrity of the intestinal barrier and facilitating translocation of bacterial endotoxins into the bloodstream, consequently triggering a systemic immunological response [38]. Bansal et al. [39] observed that indole compounds from gut microbiota reduce inflammatory factor production and safeguard intestinal wall integrity, and this conclusion reinforced by in vivo research by Shimada et al. [40]. Therefore, it is plausible that 3-indoleglyoxylic acid secreted by Ruminococcus2 may contribute to reducing the incidence of NAFLD through its ability to protect intestinal wall integrity and suppress the production of inflammatory factors. In conclusion, this MR study provides robust and innovative evidence regarding the causal impact of gut Ruminococcus2 abundance on the risk of NAFLD. This study elucidates potential pathways mediated by cysteine-glutathione disulfide and 3-indole-glyoxylic acid levels between Ruminococcus2 and NAFLD. Future studies should prioritize examining the regulation of gut microbiota and evaluating blood metabolite levels to prevent and cure NAFLD.

Furthermore, recognizing the limits of our research is essential. First, the majority of GWAS data utilized in this study pertained to individuals of European descent, which minimizes population heterogeneity but restricts the applicability to ethnically diverse populations. As more GWAS data from other ethnic groups becomes publicly accessible, it is imperative to validate the MR results in these populations to ascertain the generalizability of the study. Second, one study suggested a possible bidirectional association between genus Ruminococcus and NAFLD [41]; however, the reverse causality was not existed in our study. This discrepancy may be attributed to variations in the demographic composition of the outcome dataset, which may influence the results obtained. It needs to be demonstrated whether NAFLD has an impact on the abundance of the genus Ruminococcus through GWAS data covering a larger population or additional experimental studies. Meanwhile, a study has demonstrated an elevated abundance of Ruminococcus in the gut microbiota of NAFLD patients, which contradicts our findings [42]. We believe that this discrepancy may be attributed to several factors. First, different research methods were used in the two studies. Our study employed the Mendelian randomization method, a form of prospective cohort study, while Jérôme Boursier’s study utilized a case-control approach. Case-control studies may be influenced by confounding factors and reverse causality. Thus, the disparity in research methodology may have contributed to divergent conclusions. Secondly, Since Jérôme Boursier’s study was conducted earlier, their definition of Ruminococcus may have differed from the current understanding. There is significant diversity within the Ruminococcus genus, with distinct subtypes of Ruminococcus exerting varying effects on the host organism [43, 44]. Our study primarily focused on the newly proposed subtype Ruminococcus2, which could contribute to the conflicting conclusions. Thirdly, Jérôme Boursier’s study had a limited sample size, consisting of only 57 NAFLD patients, with only 27 cases showing a significant association between severe fibrosis (F ≥ 2) and Ruminococcus. In contrast, the GWAS data used in our study had 4,761 cases of NAFLD. Therefore, I suspect that the main reason for the difference in results is the limitation due to the small sample size. Additionally, due to the restricted number of SNPs reaching genome-wide significance, we decided to adopt a more lenient p-value threshold. This methodology is broadly acknowledged and often utilized in research procedures [15]. Finally, while MR methods are indeed reliable for causal inference, it is crucial to acknowledge the necessity of further validation through a randomized controlled trial in order to enhance the credibility and robustness of the findings presented in this MR study.

Our study uses MR methods to minimize potential confounding effects and provides new evidence by demonstrating that genetically predicted Ruminococcus2 abundance has a direct unidirectional causal effect on NAFLD. This highlights the importance of Ruminococcus2 as a predictive and preventive factor for NAFLD risk and offers valuable insights into the potential mechanism for the gut-liver axis hypothesis. In addition, this study identified a strong association between two blood metabolites (cysteine-glutathione disulfide and 3-indole-glyoxylic acid) and the risk of NAFLD, offering new avenues for early detection and treatment of NAFLD.

## Conclusion

In conclusion, our research combines cutting-edge gut microbiota data with high-throughput metabolomics, providing a opportunity to uncover the genesis and likely causative risk factors of NAFLD using a Mendelian randomization strategy. Through a two-sample MR approach, we elucidated the involvement of two blood metabolites in mediating the causal relationship between the gut microbiota and NAFLD. Future research should aim to provide a more comprehensive understanding of the underlying mechanisms through which gut microbiota influence NAFLD via metabolites, as well as explore potential therapeutic strategies.

### Electronic supplementary material

Below is the link to the electronic supplementary material.

Supplementary Material 1

Supplementary Material 2

## Data Availability

The data of gut microbiota are openly available in MiBioGen consortium (mibiogen.gcc.rug.nl). The GWAS data of NAFLD are openly available in IEU GWAS project (gwas.mrcieu.ac.uk/). The GWAS data of NAFLD are openly available in IEU GWAS project (gwas.mrcieu.ac.uk/). The GWAS data for the 1400 metabolites were obtained from Chen et al.^11^

## Abbreviations

NAFLD: Non-Alcoholic Fatty Liver Disease
GWAS: Genome-Wide Association Study
FDR: False Discovery Rate
LDSC: Linkage Unbalanced Regression
cML-MA: Constrained Maximum Likelihood and Model Averaging
OR: Odds Ratio
HCC: Hepatocellular Carcinoma
MR: Mendelian Randomization
IVs: Instrumental Variables
rRNA: Ribosomal Ribonucleic Acid
IEU: Integrative Epidemiology Unit
SNPs: Single Nucleotide Polymorphisms
SE: Standard Error
WM: Weighted Median
IVW: Inverse Variance Weighted
FMT: Fecal Microbiota Transplantation
LDL: Low-Density Lipoprotein
