# Supplementary material for: Metabolites mediate the causal associations between gut microbiota and NAFLD: a Mendelian randomization study
Source: BMC Gastroenterol. 2024 Jul 31;24:244. doi: 10.1186/s12876-024-03277-w (PMC11292861; doi:10.1186/s12876-024-03277-w)
Supplement: Supplementary file 1 — Supplementary Material 1 [file 12876_2024_3277_MOESM1_ESM.docx]

Supplementary Figure 1


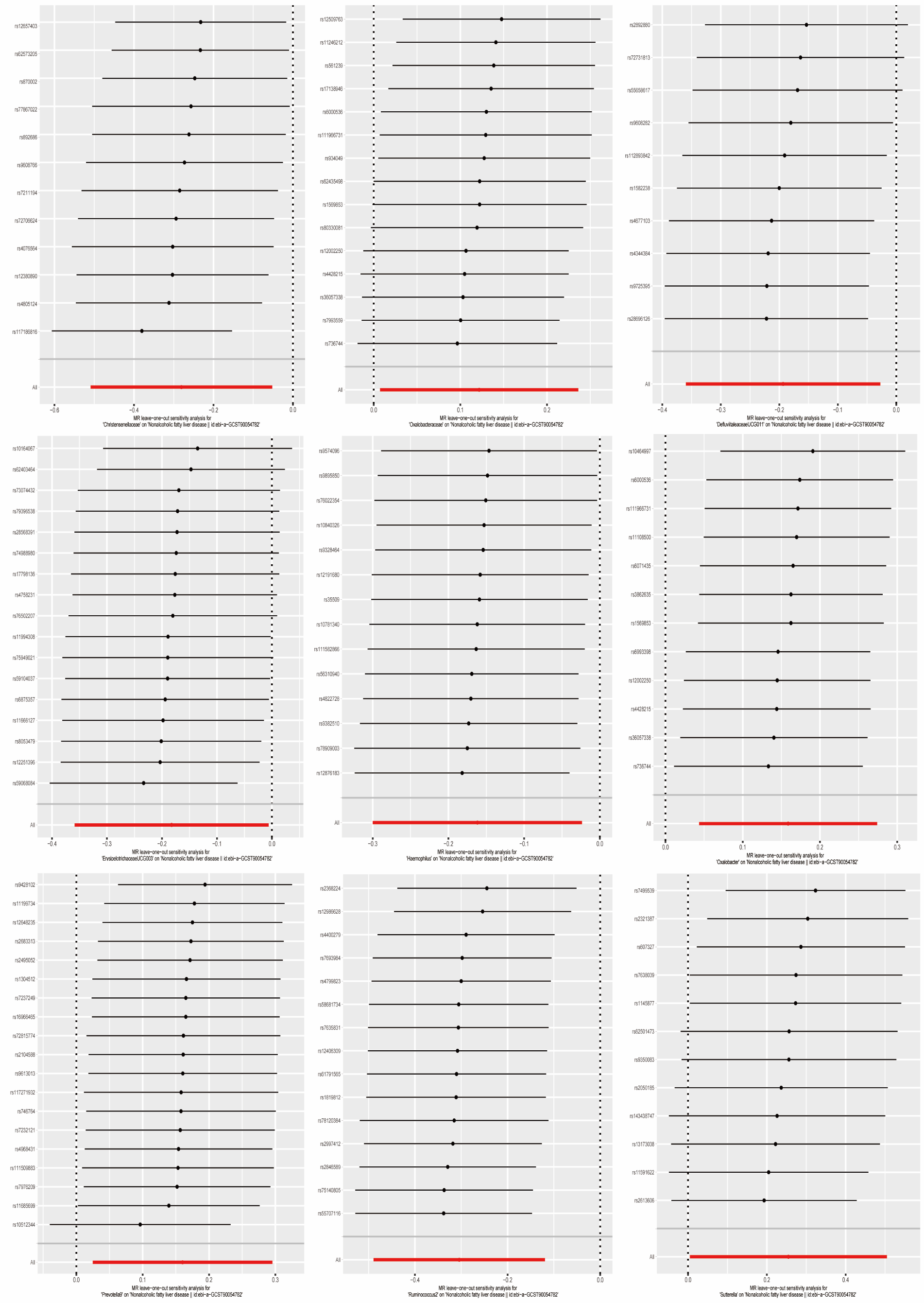


The results of leave-one-out analysis for nine gut microbiotas associated with NAFLD.

Supplementary Figure 2


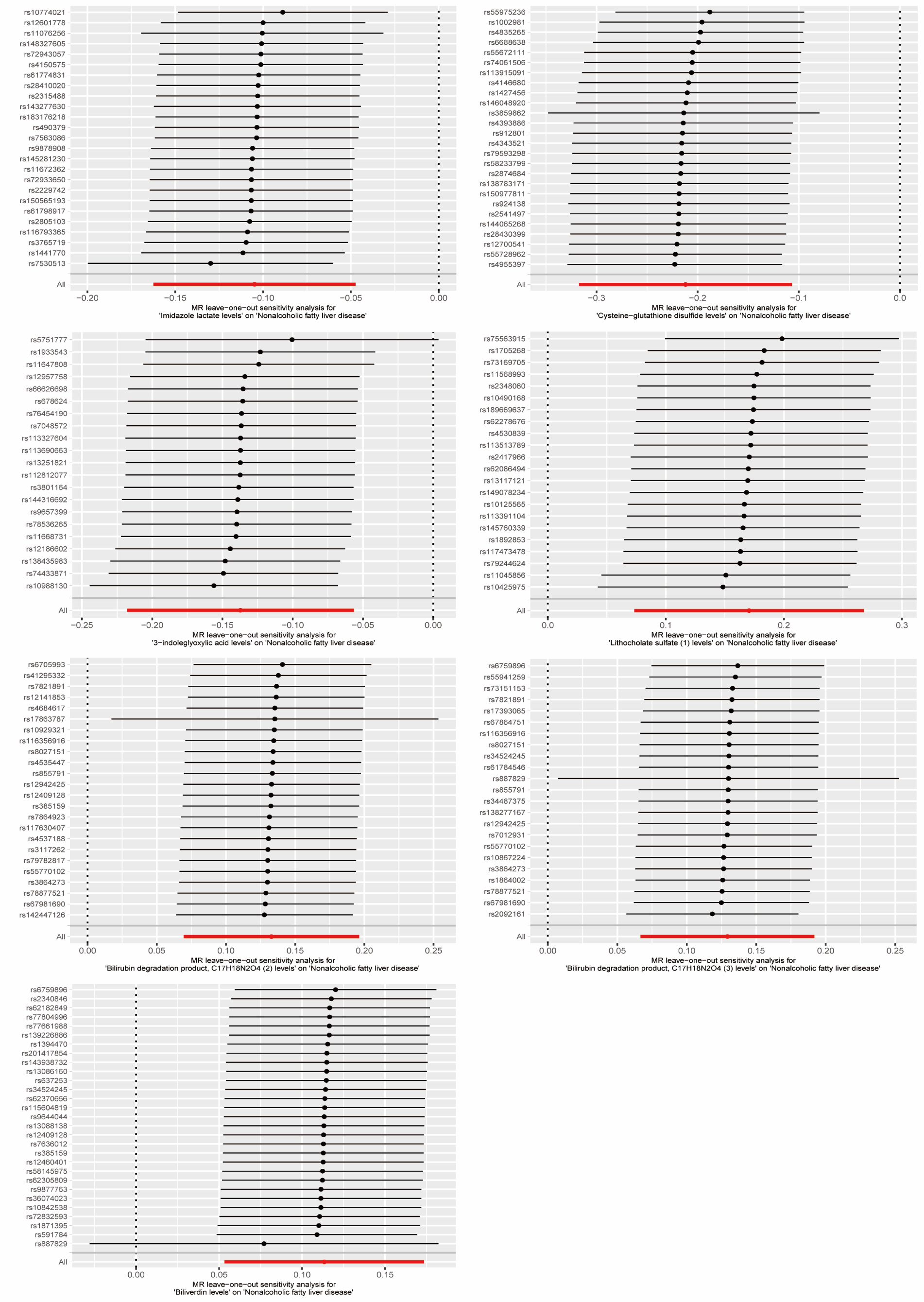


The results of leave-one-out analysis for seven blood metabolites associated with NAFLD.

Supplementary Figure 3


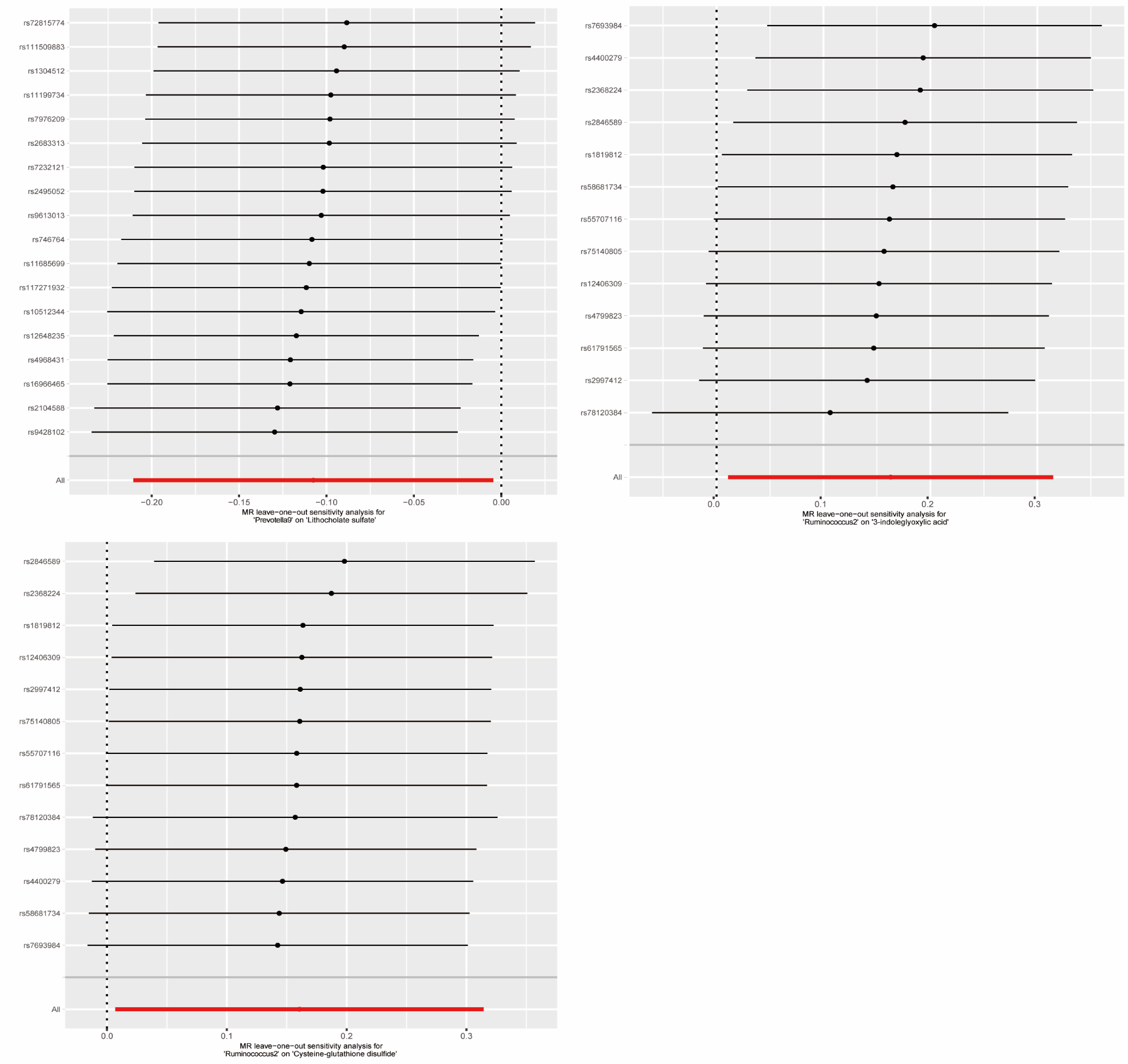


The results of leave-one-out analysis for three gut microbiotas associated with metabolites.

Supplementary Figure 4


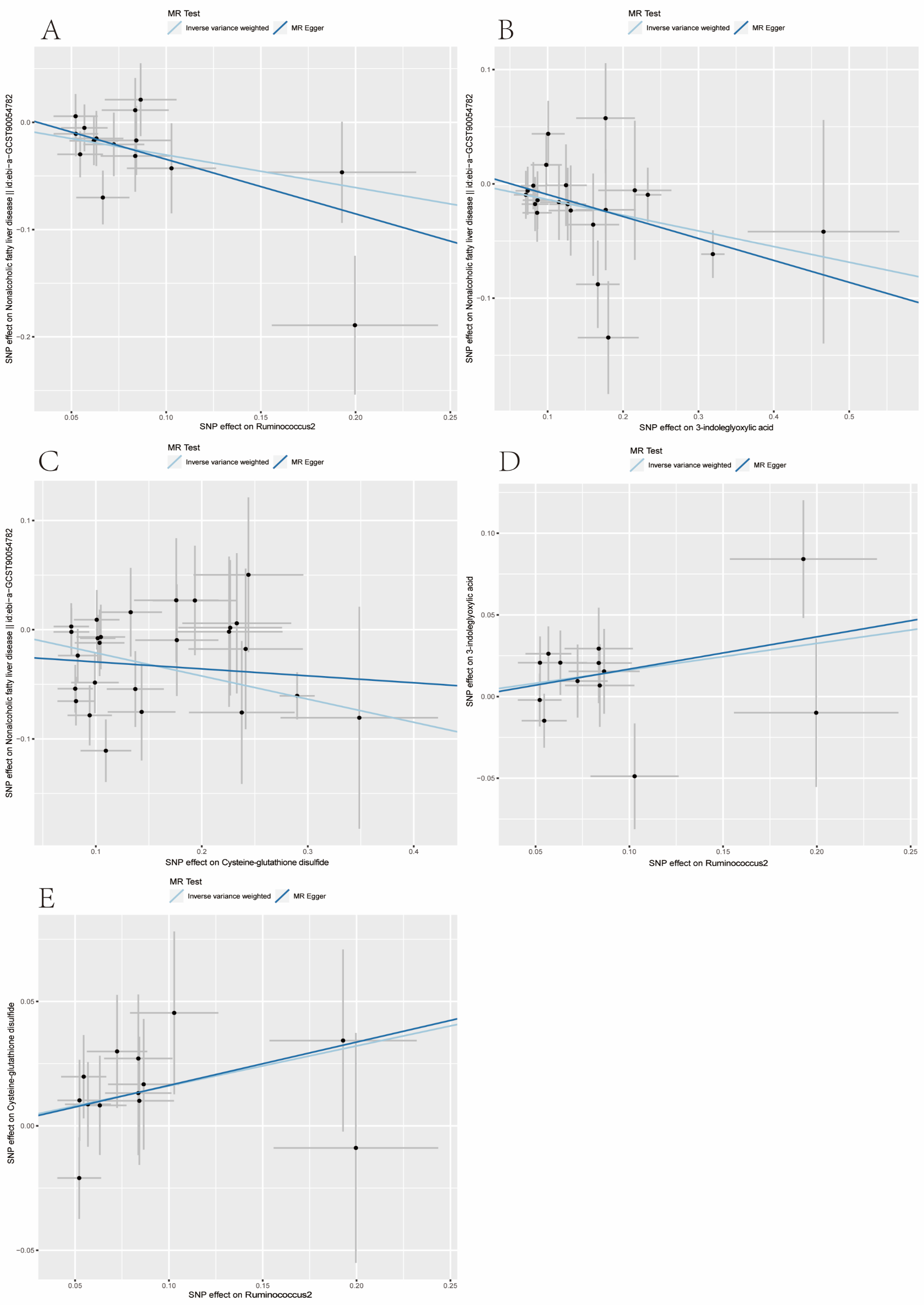


The scatter plots of the MR results (A) the Mendelian randomization results of the gut microbiota genus Ruminococcus2 with NAFLD; (B) the Mendelian randomization results of the 3-indoleglyoxylic acid with NAFLD; (C) the Mendelian randomization results of the Cysteine-glutathione disulfide with NAFLD; (D) the Mendelian randomization results of the Ruminococcus2 with 3-indoleglyoxylic acid; (E)the Mendelian randomization results of the Ruminococcus2 with Cysteine-glutathione disulfide.
